# Math Anxiety Is Related to Some, but Not All, Experiences with Math

**DOI:** 10.3389/fpsyg.2017.02067

**Published:** 2017-12-01

**Authors:** Krystle O'Leary, Cheryll L. Fitzpatrick, Darcy Hallett

**Affiliations:** Psychology, Memorial University of Newfoundland, St. John's, NL, Canada

**Keywords:** math anxiety, math experiences, math cognition, anxiety, educational psychology

## Abstract

Math anxiety has been defined as unpleasant feelings of tension and anxiety that hinder the ability to deal with numbers and math in a variety of situations. Although many studies have looked at situational and demographic factors associated with math anxiety, little research has looked at the self-reported experiences with math that are associated with math anxiety. The present study used a mixed-methods design and surveyed 131 undergraduate students about their experiences with math through elementary school, junior high, and high school, while also assessing math anxiety, general anxiety, and test anxiety. Some reported experiences (e.g., support in high school, giving students plenty of examples) were significantly related to the level of math anxiety, even after controlling for general and test anxiety, but many other factors originally thought to be related to math anxiety did not demonstrate a relation in this study. Overall, this study addresses a gap in the literature and provides some suggestive specifics of the kinds of past experiences that are related to math anxiety and those that are not.

## Introduction

Math skills are essential for individuals' participation in society and success in everyday life (Maloney et al., 2010). Yet, many individuals have a fear of math and numbers, commonly referred to as math anxiety (Beilock and Maloney, 2015). Math anxiety (MA) is a negative response experienced by some individuals when they are faced with numbers, math, and calculations (Ashcraft and Moore, 2009). Richardson and Suinn (1972) defined math anxiety as “unpleasant feelings, specifically, those of tension and anxiety that impede an individual's ability to manipulate numbers and solve math problems in a variety of situations” (p. 551)—ranging from those in a classroom setting to those encountered in everyday life (Ashcraft and Moore, 2009).

Factors shown to be associated with MA can be grouped into three broad categories: situational, dispositional, and environmental (Byrd, 1982; Baloglu and Koçak, 2006). Situational factors are those directly associated with math (Fitzgerald, 1997) including the construct itself (in this case math) as well as variables surrounding the construct (Byrd, 1982). Dispositional factors are personality factors that make an individual more likely to experience math anxiety and can be considered a vulnerability to math anxiety (Baloglu and Koçak, 2006). Finally, environmental factors consist of an individual's previous experiences with and perceptions of math (Baloglu and Koçak, 2006).

Although all of these factors contribute to MA, the current study examined a particular type of environmental factor—math experiences. Other research has examined the influence of environmental factors, like having a teacher that is math anxious (Beilock et al., 2010) or having a parent who is math anxious (Maloney et al., 2015)—findings that imply that being exposed to negative attitudes of math may lead to math anxiety (Beilock and Maloney, 2015). The current study, however, specifically examines people's memories about their experiences with math, or, in other words, their self-reported math experiences. There have been a few studies that have looked at self-reported experiences with math (e.g., Jackson and Leffingwell, 1999; Zopp, 1999; Brady and Bowd, 2005; Schmidt, 2005) which offer some suggestive results. However, these studies also suffer from various methodological shortcomings which impact the conclusions which can be drawn. The majority of the research conducted in this area exhibits one or more of the following problems: (1) only assessing self-reported experiences of math teachers or pre-service teacher; (2) sampling only math-anxious individuals; or, (3) not controlling for general anxiety.

The purpose of this study is to more rigorously test the conclusions of these previous studies concerning which experiences are related to math anxiety. Despite some of the shortcomings in the current line of literature, there are common themes that arise in this research that are worth investigating. It stands to reason that certain kinds of negative experiences could lead somebody to be more math anxious and certain kinds of positive experiences might make one less likely to have math anxiety, but they may not necessarily be the ones reported in previous research. This study attempts to shed light on what some of these experiences could be.

## Math experiences

Among math experiences, the relation between MA and math instructional practices has been examined most often in the literature (Harper and Daane, 1998; Jackson and Leffingwell, 1999; Brady and Bowd, 2005) and tends to focus on the experiences of pre-service teachers (Jackson and Leffingwell, 1999; Brady and Bowd, 2005), who typically report that their own teachers' behaviors contributes to their experiences of MA. Jackson and Leffingwell (1999) asked 157 pre-service teachers to respond to the following question: “Describe your worst or most challenging mathematics classroom experience from kindergarten through college.” In addition to this, they were asked to describe what could have been done to bring about more positive math experiences. Their results suggested a number of behaviors shown by teachers while teaching math to be related to a student's anxiety around the subject. These behaviors include embarrassing students in front of their classmates (e.g., by making negative comments toward them or by making their mistakes known to the entire class), showing signs of gender bias, having a negative attitude, responding angrily when asked for clarification, and showing a lack of understanding for those who needed extra time to grasp difficult math concepts. Jackson and Leffingwell also reported a link between perceived teacher personality types and math anxiety. Individuals who reported higher math anxiety were more likely to report that their teachers behaved in a manner that was hostile, insensitive, impatient, and critical.

Similarly, Brady and Bowd (2005) found that negative experiences in elementary and secondary school with math instructors was one of two main contributors to math anxiety (the other being the highest level math course taken). Their study examined the relationship between math anxiety, formal math education, attitudes toward math, and past math experiences in a group of pre-service teachers. The findings are noteworthy as participants consistently reported instructional methods were related to their math anxiety, regardless of the fact that the instrument used did not specifically ask about those experiences. Examples of instructional methods that reportedly hindered participants' ability to learn math included teaching at a fast pace that they could not keep up with and being made to feel unintelligent (i.e., when asking for help or stating they did not understand). Brady and Bowd also found that math anxiety and negative math instruction experiences had a great influence over pre-service teachers' confidence in teaching math.

Other studies investigating self-reported math experiences point to a relation between MA and a broader range of experiences beyond instructional methods. Schmidt (2005) conducted a qualitative study on math anxious college student's math experiences and eight major themes emerged: (1) disrespecting, humiliation, and fear-based instruction; (2) disbelieving or abusive parents, turbulent home life, and parental conflict; (3) major life transitions; (4) math-me inadequacy and negative self-appraisal; (5) perfectionism; (6) culture and gender; (7) acceptance to hate and or flunk math; and (8) respectful and supportive instruction. Similar themes have emerged from other research. Specifically, Zopp (1999) questioned eight adult learners from a larger group of 135 adults who scored high on MA. These participants indicated specific events in their education, life events (i.e., changing schools and working while attending school), and lack of support as contributing to MA. These are some of the same themes found by others in the literature (Jackson and Leffingwell, 1999; Brady and Bowd, 2005). The caveats in the methodology used in all of the above-mentioned studies (Jackson and Leffingwell, 1999; Zopp, 1999; Brady and Bowd, 2005; Schmidt, 2005), is that of generalizability, as all of the participants were highly math-anxious (pre-service teachers are known to have high MA, Dew et al., 1983; Hembree, 1990), so there is no way to tell if these experiences are what differentiate non-math-anxious individuals from math-anxious individuals.

There are a limited number of studies that have included participants with a range of levels of MA in adult (Hunsley and Flessati, 1988; Flessati and Jamieson, 1991) and child populations (Bonnstetter, 2007). Hunsley and Flessati (1988) pitted the sex role hypothesis against the math experience hypothesis (for detailed explanations, see Hunsley and Flessati, 1988) as a way to explain the gender difference sometimes seen in MA. Their results supported the math experience hypothesis, specifically, individuals who experienced the highest levels of MA also had the least amount of math experience, lowest math grades, and the highest levels of negative beliefs about math. These findings were further supported through a replication of Hunsley and Felssati's work (Flessati and Jamieson, 1991), however, these results need to be interpreted cautiously as there appeared to be a large amount of missing data.

Bonnstetter (2007) interviewed 11 children, ranging from fourth to eighth grade, comparing their levels of MA with what they reported in a previous study (Bonnstetter, 1999, as cited in Bonnstetter, 2007). The same six themes emerged, namely, feelings about self, feelings about math, concepts in math, instructional/learning style, teacher characteristics, and teacher strategies. These themes correspond to some of themes which have emerged throughout the literature (e.g., Jackson and Leffingwell, 1999; Zopp, 1999; Brady and Bowd, 2005; Schmidt, 2005). Individuals who were math-anxious had more negative experiences with regards to the six themes than non-math-anxious individuals. One of the main limitations of this study was the small sample size and therefore, it is difficult to discern whether similar results would be obtained with a larger sample and if the results can be generalized to other populations.

It is also worth noting that none of the above research controlled for general anxiety when measuring math anxiety, so it is not certain if the reported experiences are unique to math anxiety or just related to anxiety in general. Previous research has shown math anxiety to be correlated with test anxiety (Dew et al., 1983) and this notion has been further supported through a meta-analysis conducted by Hembree (1990) who found test anxiety to be highly correlated with math anxiety. Nevertheless, only 37% of one construct can be predicted from the other, indicating they are indeed separate, but related, constructs. It is important when studies are examining math anxiety that they also control for important associated factors such as general and test anxiety as they have been found to be related to math anxiety, as it is possible for an individual to experiences anxiety surround testing that is not specific to math but instead is experienced in all testing situations. Without controlling for these factors on the surface, general and test anxieties may be misrepresented as math anxiety, thus inflating the commonness of math anxiety.

## The current study

Despite their weaknesses, the literature reviewed above does provide useful suggestions about how personal experiences with math are related to MA. First, methods of instruction appear to be related to the development of MA, with math-anxious individuals consistently reporting problems with the teaching methods they were exposed to. Second, individuals with high MA commonly report a lack of support by parents and teachers. Third, math-anxious individuals commonly report experiencing negative life events (e.g., changing schools, moving houses, divorce, and mental health issues in the family) and having negative feelings about themselves and math. Lastly, performance in math influences levels of MA, in that those with lower math grades tend to have higher levels of MA. As these four themes continually emerge throughout the literature, the current study focused on assessing the potential relation of these themes with MA. As there is no known measure of math experiences that looks specifically at the variables identified above, one of the objectives of the present study was to create a measure examining these variables that could also be used as a stepping-stone for future research.

These relations were also retroactively assessed during primary education (e.g., Elementary, Junior High, and High School), as existing research now highlights the development of MA and its possible impact on younger children (Maloney and Beilock, 2012). Asking about math-related experiences during different educational periods could further the understanding of MA and whether different kinds of experiences may have different influences at different levels of schooling. Furthermore, previous research has found differences in the kinds of experiences reported during different schooling periods (e.g., Jackson and Leffingwell, 1999; Brady and Bowd, 2005).

Given the research reported above, we hypothesize that, for each of the proposed areas of experience identified (i.e., lack of support, poor/negative experiences with instructional methods, more negative life events, and low math marks) participants who self-reported experiencing more of these will have higher MA than those who did not have these experiences. Nevertheless, given the methodological flaws with the previous research that identified these themes, we expect some of these hypotheses to be rejected or qualified. In addition, the current study used a wide range of math-anxious and non-math-anxious individuals. We also measured general and test anxiety to evaluate whether the relation between math experiences and MA exists beyond general and test anxiety.

## Methods

### Participants

We sampled 131 undergraduate students who received course credit for participating: 34 (26%) were male and 94 (73%) were female; one person did not indicate their gender. Students ranged from 18 to 41 years-of-age (*M* = 21.80, *SD* = 3.71). Declared majors included backgrounds in Psychology (*n* = 57, 44%), other areas of Science (*n* = 40, 31%), other areas in Arts (*n* = 18, 14%), Education (*n* = 1, 0.78%), and no major declared (*n* = 7, 5.5%). Most students were in their second year of university (*n* = 50, 38.2%), followed by third year (*n* = 34, 26%), first year (*n* = 25, 19.1%), fourth year (*n* = 17, 13%), and unknown year of study (*n* = 5, 3.8%). The majority of our sample was Caucasian (*n* = 107, 81.7%) and the remaining minority consisted of Aboriginal, Asian, and Indian (*n* = 4, 3.1%), Southeast Asian and Hispanic (*n* = 1, 0.8%), and 7 of unknown ethnicity (5.3%).

### Measures

All of the measures in this study were administered in paper-and-pencil format.

#### Demographic questionnaire

This questionnaire was used to gather relevant demographic information and was composed of short fill-in-the blank questions, assessing gender, age, ethnicity, and academic information (e.g., year of degree, major, or minor).

#### Math anxiety rating scale-short version (MARS-S; Suinn and Winston, 2003)

The MARS was the first instrument constructed to measure individual's level of MA (Ashcraft, 2002) and is one of the most commonly used scales to measure MA (Wigfield and Meece, 1988). The MARS-S was created by drawing 30 items from the original 98-item version measure. Examples of items include: “taking an examination (quiz) in a math course,” “listening to a lecture in a math course,” “totaling up a dinner bill that you think you were overcharged on,” and “calculating the sales tax on a purchase that costs more than $1.00.” This measure can be administered in an individual or group setting and respondents are asked to rate their level of anxiety on a 5-point Likert scale (1-not at all to 5-very much). Items are summed together, obtaining a total score ranging from 30 to 150, with higher scores indicating higher levels of MA. One-week test-retest reliability for college students has been found to be 0.90, which is quite similar to that of the MARS 98-item measure. Participants' levels of MA ranged from 30 to 148 (*M* = 68.40, *SD* = 18.82), suggesting our sample represented a range of math anxious and non-math anxious individuals. Preliminary analyses show that the Psychology majors had higher math anxiety, *M* = 70.4, *SD* = 19.0, than those from other areas of Science, *M* = 59.8, *SD* = 17.2, but did not differ from those in other areas of Arts, *M* = 76.6, *SD* = 18.1, *F*_(2, 112)_ = 6.54, *p* = 0.002, $\eta_{\text{p}}^{2}$ = 0.105.

#### International personality item pool (IPIP; Goldberg, 1999)

The IPIP's representation of Costa and McCrae's (1992) five NEO domains is a 50-item self-report questionnaire. It measures the five domains of personality: neuroticism, extraversion, openness to experience, agreeableness, and conscientiousness. This measure has an internal consistency of 0.80. Data from this measure were not analyzed in this study.

#### Test anxiety inventory (TAI; Spielberger, 1980)

The TAI is a 20-item self-report questionnaire developed to assess test anxiety. It can be administered in an individual or group setting, and takes approximately 8–10 min to complete. Respondents are asked to rate how often they are troubled with particular symptoms of anxiety during, after, and before a test on a 4-point Likert scale (1-almost never to 4-almost always). Items are summed together, obtaining a total score ranging from 20 to 80; higher scores indicate higher levels of test anxiety. Normative data is based on 1,449 undergraduate students. Two-week and three-week test-retest reliability for a college sample has been found to be 0.80. Internal consistency has ranged from 0.61 to 0.69. The participants' level of test anxiety as measure by the TAI ranged from 20 to 79 (*M* = 43.68, *SD* = 13.58).

#### Penn state worry questionnaire (PSWQ; Meyer et al., 1990)

The PSWQ is a 16-item self-report questionnaire assessing level of worry. Respondents are asked to rate how typical it is for them to experience worrisome thoughts on a 5-point Likert scale (1-not at all typical of me to 5-very typical of me). Items are summed together, obtaining a total score ranging from 16 to 80 with higher scores indicating higher levels of worry. The PSWQ has a high test-retest reliability of 0.92 and an internal consistency of 0.94. Participants' level of anxiety as measure by the PSWQ ranged from 20.96 to 80 (*M* = 54.68, *SD* = 14.16). This measure was included as one of two measures to control for general anxiety.

#### Beck anxiety inventory (BAI; Beck et al., 1988)

The BAI is a 21-item self-report questionnaire developed to assess an individual's level of anxiety. Individuals are asked to rate how often they are troubled by a symptom during the past month on a 4-point Likert scale (0-not at all to 3-severely, it bothered me a lot). Participants' level of anxiety as measured by the BAI ranged from 0 to 47 (*M* = 17.25, *SD* = 10.81). The BAI has an internal consistency ranging from 0.92 to 0.94 for adults and a 1-week test-retest reliability of 0.75. This measure, in addition to the PSWQ, was included to control for general anxiety.

### Development of the math experience questionnaire

This measure was created specifically for the current study to gain insight into individuals' experiences with math. For the purpose of this research, *math experience* is defined as a personal event or situation, encountered or perceived, involving math. Items for this measure were either derived from other measures of math experience (e.g., Zopp, 1999; Schmidt, 2005) or were created by the researchers. Once a large pool of items were created, members of our research lab examined the items to ensure their clarity, that they accurately reflected the construct being examined, that the language was appropriate, and that there were no double-barreled items. The measure is composed of both Likert-type questions as well as open-ended questions. Open-ended questions were included to ensure that no vital information was being missed. The final version of the measure has a grade reading level of 7.2, as reported by Microsoft Office.

The first part of this measure is composed of 72 Likert-items, rated on a 5-point scale, ranging from 1-strongly agree to 5-strongly disagree, while the second part had 14 open-ended questions (see Appendix B in Supplementary Material). The 72 Likert-items are 24 items repeated three times, to separately reflect experiences in Elementary School (Grades 4–6), Junior High (Grades 7–9), and High School (Grades 10–12). These three levels of schooling were chosen because previous research has found differences in math experience between different levels of schooling (Jackson and Leffingwell, 1999; Brady and Bowd, 2005) and because these particular grade levels reflected how schools were divided in the local area. The Likert-items measured three of the four themes identified above: (1) Support; (2) Instructional Methods; and (3) Math Marks (see Appendix A in Supplementary Material for full list of items). The three levels of schooling were highly correlated within both the Support and Instructional Methods subscales (*r*s range from 0.325 to 0.525), but were not highly enough correlated to suggest that participants did not differentiate between these different levels of schooling. For the math marks subscale, the correlations were higher (*r*s range from 0.600 to 0.724), but it might be expected that participants memories of their marks were more consistent across the different levels of schooling as their marks likely were fairly consistent.

Additional items were added that individually assessed gender-related experiences (items 3 and 15), calculator use when working on math problems (item 20), and their teacher's attitude about math (item 23). None of these miscellaneous items were related to MA, so no further analyses were conducted with them. The 14 open-ended questions mostly examined the life events theme, however, there were some open-ended questions that assessed both the support and the instructional methods theme. The open-ended questions were included to ensure that no vital information was being missed. Consequently, the information gleaned from these open-ended responses can serve to improve upon the types of Likert-items that should be included in a questionnaire that measures a person's math experiences. Unlike the Likert-items, the 14 open-ended questions were only given once at the end of the MEQ, and were not asked three times to reflect the different levels of schooling. This was done to avoid making the measure too onerous and to reduce measurement fatigue.

### Reliability analyses

Following data collection, all Likert-items were assessed for internal consistency. Items were candidates for elimination if they approached near zero or negative item-total correlations and if their elimination increased the internal consistency of the scale. Although reliability analyses were completed separately for each scale, we wanted each scale to consist of the same items across the different levels of schooling (i.e., Elementary, Junior High, and High School). The results of these analyses were fairly consistent across the different educational periods for all dimensions. There were four items in the support scale, one item (item 18) met elimination criteria for all levels of schooling and was removed from the scale. A second item (item 16) met these criteria for Elementary School, but the removal of this item would have noticeably decreased the reliability of the Junior High and High School scales, so it remained in the final scale. The resulting three-item scale had internal consistencies of 0.79, 0.77, and 0.85 for Elementary, Junior High and High School, respectively.

The instructional scale was composed of 13 items; however, three items (items 4, 17, and 21) met the elimination criteria for all levels of schooling and were removed from the scales. A fourth item (item 19) met the elimination criteria for Junior High and High School, but not for Elementary School. This item was removed because it increased the reliability of the scale for the Junior High and High School levels considerably and did not greatly reduce the reliability for Elementary School. The final nine-item scale had an internal consistency of 0.83, 0.86, and 0.88 for Elementary, Junior High, and High School, respectively.

In the case of the math marks scale, there were three items. One item (item 24) met the elimination criteria for Elementary School. However, the removal of this item posed a substantial decrease in reliability for the other levels of schooling and only a minimal increase in reliability for the Elementary School level. All three items remained in the scale and had an internal consistency of 0.91, 0.92, and 0.93 for Elementary, Junior High, and High School, respectively.

The final version of this measure was composed of 51 Likert-items and is described in Appendix A (Supplementary Material).

### Procedure

Participants were recruited through undergraduate psychology courses at a Canadian university and were given course credit for their participation. Students completed a paper-and-pencil format questionnaire packet containing the MARS-S, the Math Experience Questionnaire, the IPIP (Goldberg, 1999), the TAI, the PSWQ, the BAI, and a demographics questionnaire. The MARS and Math Experience Questionnaire were included to test the primary hypotheses of the study while the last three measures were included so that MA could be examined while statistically controlling for test anxiety (TAI) and general anxiety (PSWQ and BAI). Data from the IPIP test were not analyzed in this study. Questionnaire order was counterbalanced using a Latin square design, using six different ordering sequences, to prevent order effects. The questionnaire packet took participants approximately 60–90 min to complete. Participants were debriefed upon completion and thanked for their time. Prior to data collection, study procedures had been reviewed and approved by the local Institutional Ethics Review Board.

## Results

Prior to data analysis, demographic and questionnaire data were examined for missing data points, outliers, and normality. Missing data, although rare, were excluded in a pairwise fashion. There were two outliers, one on the BAI (*z* = 3.32) and the other on the MARS (*z* = 3.94); these participants were removed from the sample. The BAI, TAI, and MARS are all positively skewed (see Table 1), but the level of skew is not so great that it would have an effect on correlations (Dunlap et al., 1995). As expected, MA was moderately correlated with the BAI, TAI, and PSWQ (see Table 2).

**Table 1:** Participants' Mean Scores and Standard Deviation on the Math Anxiety Rating Scale-Short Version (MARS-S), the Test Anxiety Inventory (TAI), Beck Anxiety Inventory (BAI), and the Penn State Worry Questionnaire (PSWQ) (*N* = 129).

**Measure (*N*)**	**Mean (*SD*)**	**Skew (*SE*)**	**Kurtosis (*SE*)**
MARS-S	68.42 (18.82)	0.166 (0.213)	−0.615 (0.423)
TAI	43.68 (13.57)	0.669 (0.213)	−0.124 (0.423)
PSWQ	54.68 (14.16)	−0.343 (0.213)	−0.816 (0.423)
BAI	17.25 (10.81)	0.739 (0.214)	−0.109 (0.425)

**Table 2:** Correlations between Math Anxiety, Test Anxiety, and General Anxiety as measured by the Beck Anxiety Inventory and Penn state Worry Questionnaire (*N* = 129).

	**Math anxiety**	**Test anxiety**	**Beck anxiety inventory**	**Penn state worry questionnaire**
Math anxiety	1			
Test anxiety	0.687^**^	1	–	
Beck anxiety inventory	0.566^**^	0.607^**^	1	–
Penn state worry questionnaire	0.411^**^	0.504^**^	0.536^**^	1

** *p < 0.01*.

Math experience was measured using Likert-items and open-ended questions. The responses to the 14 open-ended questions were coded and analyzed by the lead researcher [all codes are listed in Appendix B (Supplementary Material)], and a second coder analyzed a random selection of 20% of questionnaires using the same coding scheme. The percentage occurrence agreement between the two coders was 83% (See Appendix B in Supplementary Material for the percentage occurrence agreement per question). Disagreements between the coders were resolved by discussion. Although this inter-rater agreement does not take into account chance agreement, as would a kappa statistic, the probability of chance agreement is fairly low, as there are a large number of codes for each question (between 6 and 15 codes) and a total of 135 codes across all questions. Moreover, each question could be coded as more than one code, which means that a kappa can only be calculated for each of the 135 codes, and not for each question overall. Across these 135 codes, two-thirds had 100% percent agreement, and all but 5 of the codes had a kappa above 0.5. In sum, the coding scheme had good inter-rater reliability.

Analyses are described separately based on the themes of Support, Instructional Methods, Life Events, and Math Marks. For the open-ended questions, each question was analyzed by first comparing differences in MA between people who answered that particular question in any affirmative way and those who did not. This was called the general response, and it was coded as either Yes or No. This was followed up by testing differences in MA between those who gave specific responses and those that did not answer the question in any affirmative way (i.e., the No group), provided there were at least 10 participants in each group. This was done even in cases where no significant difference was found in MA for the general Yes/No responses to examine the possibility of MA differences only existing for more specific experiences. The one exception to this was Question 10, which asks what experiences in school affected feelings about math. It made no sense to aggregate general responses for this question because some answers related positive experiences, some answers related negative experiences, and only six people related no experience at all. Instead, each specific response was compared to everybody who did not make that response. All means, standard deviations, group sizes, and *t*-test information are presented in **Tables 4**–**6** for the Support, Instructional Methods, and Life Events dimensions, respectively.

Because of the multiple tests, an alpha of 0.01 was used for all significance tests. This alpha was chosen both to account for the possible inflation of Type I error while simultaneously not inflating Type II error to the point that would drastically curtail the interpretability of the data. Furthermore, any results that were significant or trending (i.e., *p* < 0.05) were followed up to determine if the effects remained or became significant after controlling for the influence of general and test anxiety. While the results of the initial *t*-tests are reported in **Tables 4**–**6** (with statistical significance marked by a single asterisk), the follow-up analyses controlling for general and text anxiety are reported in the text (but significant findings are marked on **Tables 4**–**6** with a double asterisk).

### Support

Bivariate correlations assessed the relation between MA, measured by the MARS, and the Support dimension, measured by the Math Experience Questionnaire. Support at all levels of schooling were significantly and negatively related to MA. Semi-partial correlations controlled for general and test anxiety (see Table 3), and only experiences with regards to Support in High School remained related to MA.

**Table 3:** Correlations between Math Anxiety and Support, Instructional Methods, and Math Marks across the three levels of schooling (*N* = 129).

	**Math anxiety**	**Math anxiety controlling for general and test anxiety**
Support Elementary School	−0.236^**^N = 127	−0.123 *df* = 118
Support Junior High School	−0.230^**^N = 129	−0.122 *df* = 118
Support High School	−0.269^**^N = 129	−0.317^**^df = 118
Instructional Methods Elementary School	−0.338^**^N = 128	−0.214^*^df = 120
Instructional Methods Junior High School	−0.267^**^N = 129	−0.167 *df* = 118
Instructional Methods High School	−0.248^**^N = 129	−0.270^**^df = 118
Math Marks Elementary School	−0.441^**^N = 126	−0.435^**^df = 118
Math Marks Junior High School	−0.515^**^N = 128	−0.424^**^df = 118
Math Marks High School	−0.475^**^N = 128	−0.430^**^df = 118

* *p < 0.05*.

** *p < 0.01*.

Responses to the three open-ended questions related to the Support dimension (see Table 4) demonstrated only a few differences in MA. For the general responses, there was only a significant difference in MA for those who reported having someone decrease their math confidence. For the specific responses, those who said a teacher made them feel poorly about themselves reported significantly higher MA than those who reported no one did something to decrease their math confidence, and participants who reported being involved in math competitions trended toward lower MA than those who did not. However, once general and test anxiety were accounted for, all of these relations no longer existed [*M*_*Diff*_ = 4.70, *t*_(122)_ = 1.88, *p* = 0.063, Cohen's *d* = 0.36, *M*_*Diff*_ = 8.42, *t*_(65)_ = 2.08, *p* = 0.042, Cohen's *d* = 0.59, and *M*_*Diff*_ = 1.18, *t*_(21)_ = −0.20, *p* = 0.846, Cohen's *d* = 0.11, respectively]. In contrast, those who experienced help from their parents had a tendency toward lower MA compared to those who did not, and this difference became statistically significant once general and test anxiety had been accounted for, *M*_*Diff*_ = −8.97, *t*_(38)_ = −2.76, *p* = 0.009, Cohen's *d* = −0.61. No other responses demonstrated a difference in MA.

**Table 4:** Math Anxiety differences with responses provided by 10 or more participants on the Support Scale Open-ended Questions.

**Open-ended question response**	**Mean (*SD*)**	***t*-value (*df*)**	***p*-value**
**1. SOMEONE DID SOMETHING TO INCREASE MATH CONFIDENCE**			
No (*n* = 14, 10.9%)^†^	73.07 (16.09)		
Yes (*n* = 114, 88.4%)	67.79 (19.18)	−0.99(126)	0.325
Encouragement from a teacher (*n* = 38, 29.5%)	62.47 (18.26)	−1.91(50)	0.062
Receiving extra help from a teacher (*n* = 20, 15.5%)	73.93 (19.90)	0.13(32)	0.895
Encouragement from a parent (*n* = 20, 15.5%)	62.87 (23.96)	−1.39(32)	0.175
Receiving extra help from a parent (*n* = 17, 13.2%)	69.67 (19.20)	−0.53(29)	0.602
Being asked to tutor others (*n* = 15, 11.6%)	68.47 (20.41)	−0.67(27)	0.508
Being Involved in math enrichment (*n* = 14, 10.9%)	61.78 (19.66)	−1.66(26)	0.108
Being involved in Math competitions (*n* = 12, 9.3%)	56.33 (15.46)	−2.69(24)	0.013
**2. SOMEONE DID SOMETHING TO DECREASE MATH CONFIDENCE**			
No (*n* = 51, 39.5%)^†^	61.86 (18.40)	3.54 (126)	0.001^*^
Yes (*n* = 77, 59.7%)	73.23 (17.37)		
Having a math teacher who made them feel poorly about themselves (*n* = 20, 15.5%)	77.60 (18.10)	3.26 (69)	0.002
Lack of encouragement, praise, support, and help (*n* = 11, 8.5%)	71.36 (20.78)	1.52 (60)	0.134
**9. EVENTS AT HOME STAND OUT FOR YOU IN SHAPING YOUR FEELINGS ABOUT MATH?**			
No (*n* = 15, 11.6%)^†^	67.24 (12.07)		
Yes (*n* = 111, 86%)	68.22 (19.54)	0.19 (124)	0.850
Parents' help (*n* = 29, 22.5%)	56.83 (15.65)	−2.25 (42)	0.030^**^
Parents' encouragement and praise (*n* = 22, 17.1%)	64.34 (18.88)	0.53 (35)	0.603
Having parents or family members that were good at math (*n* = 21, 16.3%)	67.71 (21.38)	0.08 (34)	0.938
Parents stressing importance of math and to do well (*n* = 12, 9.3%)	70.75 (18.14)	0.60 (25)	0.552
Parents stressing importance to do well in general (*n* = 10, 7.8%)	64.10 (20.18)	0.49 (23)	0.630

Means, t-values, and p-value. Complete text of the open-ended questions can be found in Appendix B (Supplementary Material).

* p < 0.01.

** p < 0.01 after controlling for general and test anxiety.

† *Indicates the comparison group for all analyses within that question*.

### Instructional methods

Bivariate correlations assessed the relation between MA, measured by the MARS, and the Instructional Methods dimension, measured by the Math Experience Questionnaire. These correlations indicated that Instructional Methods were significantly and negatively related to MA during all three levels of schooling (see Table 3). Semi-partial correlations suggest that, once general anxiety and test anxiety were controlled, Instructional Methods remained correlated with MA in Elementary and High School, but not in Junior High.

At first glance, the general responses to the two open-ended questions pertaining to the Instructional Methods dimension suggest that teachers' actions have more negative relations than positive one (see Table 5). That is, there was a significant difference in MA between those who had reported a teacher doing something to increase their anxiety about math and those who did not, but there was no significant difference in MA between those who reported a teacher doing something to decrease their MA and those who did not. However, the relation for teachers doing something to increase anxiety became non-significant after controlling for both general anxiety and test anxiety, *M*_*Diff*_ = 6.04, *t*_(117)_ = 2.25, *p* = 0.027, Cohen's *d* = 0.33.

**Table 5:** Math Anxiety differences with responses provided by 10 or more participants on Instructional Scale Open-ended Questions.

**Open-ended question response**	**Mean (*SD*)**	***t*-value (*df*)**	***p*-value**
**3. TEACHER DID SOMETHING TO INCREASE MATH ANXIETY**			
No (*n* = 38, 29.5%)^†^	61.50 (16.19)		
Yes (*n* = 85, 65.9%)	71.70 (19.30)	2.84 (121)	0.005^*^
Having a teacher speak about how difficult math was (*n* = 14, 10.9%)	61.71 (17.29)	0.04 (50)	0.967
Having a math teacher who made them feel poorly about themselves (*n* = 12, 9.3%)	82.08 (18.31)	3.72 (48)	0.001^*^
**4. TEACHER DID SOMETHING TO DECREASE MATH ANXIETY**			
No (*n* = 30, 23.3%)^†^	66.81 (16.98)		
Yes (*n* = 95, 73.6%)	68.56 (19.47)	0.44 (123)	0.659
Having a teacher who was available for extra help (*n* = 32, 24.8%)	72.06 (19.60)	1.12 (60)	0.266
Having a teacher who gave encouragement, either praise, support, or both (*n* = 29, 22.5%)	71.34 (17.31)	1.02 (57)	0.314
Having a teacher who explained and/or answered questions until they were understood (*n* = 20, 15.5%)	60.10 (19.64)	1.29 (48)	0.205
Teacher provided lots of examples/practice items (*n* = 11, 8.5%)	52.36 (12.83)	2.56 (39)	0.015^**^

Complete text of the open-ended questions can be found in Appendix B (Supplementary Material).

* p < 0.01.

** p < 0.01 after controlling for general and test anxiety.

† *Indicates the comparison group for all analyses within that question*.

An examination of the more specific responses demonstrates that there were particular actions by teachers associated with increased and decreased MA, but only one that persisted after controlling for general and test anxiety. Individuals who experienced a teacher who made them feel poorly about themselves had significantly higher MA than those who did not share this experience, but this relation became non-significant after controlling for general and test anxiety, *M*_*Diff*_ = 9.76, *t*_(44)_ = 2.17, *p* = 0.036, Cohen's *d* = 0.58. On the other hand, those who reported that their teachers provided plenty of examples trended toward significantly lower MA than those who did not, and this relation became statistically significant after controlling for general anxiety and test anxiety, *M*_*Diff*_ = −13.18, *t*_(36)_ = −3.39, *p* = 0.002, Cohen's *d* = −0.82.

### Life events

The Life Events dimension addressed events at school and home that may play a role in academic performance, such as physical and mental health issues, substance use, and how a person's gender may impact their experiences in math (see Appendix C in Supplementary Material). One of the events that appears to be related to MA is the moving of homes or schools during Junior High. Nearly 50% of participants reported a move at some point during their schooling, individuals who moved homes during Junior High and those who changed schools, other than regular school level transitions, had higher MA than those who did not move (see Table 6), however, once general anxiety and test anxiety were controlled for these results became non-significant [*M*_*Diff*_ = 5.47, *t*_(122)_ = 1.42, *p* = 0.160, Cohen's *d* = 0.30, and *M*_*Diff*_ = 6.40, *t*_(122)_ = 1.69, *p* = 0.094, Cohen's *d* = 0.35, respectively]. These findings suggest that MA is not uniquely related to changing schools during Junior High. There were no other significant differences in MA for any of the open-ended questions about Life Events (see Table 6). Although there were results that trended toward significance, there was only one that became statistically significant after controlling for general and test anxiety. Participants who reported doing poorly or lower than their expectations in school had higher MA than those who did not, *M*_*Diff*_ = 9.99, *t*_(114)_ = 2.69, *p* = 0.009 Cohen's *d* = 0.54, but only after controlling for general and text anxiety.

**Table 6:** Math Anxiety differences with responses provided by 10 or more participants on Life Events Scale Open-ended Questions.

**Open-ended question response**	**Mean (*SD*)**	***t*-value (*df*)**	***p*-value**
**5. MOVING HOMES AS A CHILD AND DURING WHAT GRADES**			
Elementary School			
No (*n* = 103, 79.8%)^†^	68.19(18.41)		
Yes (*n* = 25, 19.4%)	66.06(20.45)	0.75 (126)	0.457
Junior High School			
No (*n* = 113, 87.6%)^†^	66.89(18.39)		
Yes (*n* = 14, 10.9%)	83.97(14.37)	3.35(125)	0.001^*^
Before Elementary School			
No (*n* = 103, 79.8%)^†^	68.31(18.72)		
Yes (*n* = 24, 18.6%)	70.77(19.05)	0.58 (125)	0.564
**6. CHANGE SCHOOLS OTHER THAN TRANSITIONS FROM LEVELS**			
Prior to Elementary School			
No (*n* = 104, 80.6%)^†^	68.13(19.70)		
Yes (*n* = 24, 18.8%)	69.58(15.20)	0.34 (126)	0.736
Elementary School			
No (*n* = 107, 82.9%)^†^	67.57(18.81)		
Yes (*n* = 21, 16.4%)	72.64 (19.17)	1.13 (126)	0.263
Junior High School			
No (*n* = 113, 87.6%)^†^	66.79(18.60)		
Yes (*n* = 15, 11.7%)	80.56(17.02)	−2.72 (126)	0.007^*^
**7. MOVE TO A NEW SCHOOL WITHIN THE SCHOOL YEAR**			
No (*n* = 99, 76.7%)^†^	67.05(18.58)		
Yes (*n* = 27, 20.9%)	71.78(19.69)	1.16 (124)	0.249
**8. MOVING SCHOOLS AFFECT MATH PERFORMANCE**			
No (*n* = 39, 30.2%)^†^	73.66(16.89)		
Yes (*n* = 78, 60.5%)	66.19(19.57)	2.03 (115)	0.044
**10. EVENTS IN SCHOOL THAT SHAPED FEELINGS IN MATH**			
Good math teachers (*n* = 23, 17.8%)	64.37(14.82)	0.92 (118)	0.359
Doing well and/or being confident in their math ability (*n* = 17, 13.2%)	57.88(14.78)	2.35 (118)	0.021
Being asked to take part in competitions and/or clubs (*n* = 16, 12.4%)	61.91(21.39)	1.31 (118)	0.194
Doing poorly or lower than own expectations (*n* = 15, 11.6%)	76.83(19.56)	2.06 (118)	0.042^**^
Bad math teachers (*n* = 11, 8.5%)	74.48(21.00)	−1.22 (118)	0.224
Being nominated/receiving awards for math (*n* = 11, 8.5%)	56.64(17.52)	2.06 (118)	0.042
**11. POSITIVE OR NEGATIVE EXPERIENCES IN MATH DUE TO GENDER**			
No (*n* = 101, 78.3%)^†^	68.22(19.26)		
Yes (*n* = 22, 17.1%)	67.68(17.10)	−0.12 (121)	0.905
Negative (*n* = 15, 11.6%)	72.27(16.73)	0.91 (121)	0.365
**12. DURING SCHOOL, YOU OR FAMILY MEMBER EXPERIENCE PHYSICAL OR MENTAL HEALTH PROBLEMS**			
No (*n* = 71, 55.0%)^†^	65.19(18.52)		
Yes (*n* = 55, 42.6%)	72.16(18.99)	2.07 (124)	0.040
A family member had experienced major physical health problems (*n* = 27, 20.9%)	67.91(19.10)	0.65 (96)	0.520
Personal mental health problems (*n* = 10, 7.8%)	73.90(20.56)	1.38 (79)	0.173
**13. DURING SCHOOL, YOU OR FAMILY EXPERIENCE SUBSTANCE ABUSE**			
No (*n* = 107, 82.9%)^†^	67.59(18.99)		
Yes (*n* = 16, 12.4%)	73.62(15.24)	1.21 (121)	0.228
**14. EXPERIENCES IN PERSONAL LIFE AFFECTED ACADEMIC ABILITY**			
No (*n* = 64, 49.6%)^†^	64.98(18.25)		
Yes (*n* = 63, 48.8%)	71.49(19.13)	1.96 (125)	0.052
Mental health problems, either personal or familial (*n* = 12, 9.3%)	79.00(17.13)	2.46 (74)	0.016

Complete text of the open-ended questions can be found in Appendix B (Supplementary Material).

* p < 0.01.

** p < 0.01 after controlling for general and test anxiety.

† *Indicates the comparison group for all analyses within that question*.

### Math marks

As was the case for the support and instructional scale, bivariate correlations assessed the relation between MA and the Math Marks dimensions of Math Experience. Math Marks was significantly and negatively correlated with MA, indicating that as participant's reported math grades decreased, MA increased. Semi-partial correlations indicate that, once general anxiety and test anxiety were controlled for (see Table 3) all three of these correlations remained significant. Notably, these are retrospectively recalled math marks that are self-reported and not verified. Therefore, this particular analysis assessed the relations between MA and self-reported retrospective recall of math performance.

### Summary of results

The general pattern of results is that MA was related to support, instructional methods, and math marks on the Likert scales while there were relatively few, but interesting, results with the open-ended questions. All the Likert scales across all of the school periods were negatively related to math anxiety. After controlling for general anxiety and test anxiety, the scales that remained significantly related to MA included: (1) perceived level of support in High School; (2) instructional methods in Elementary and High School; and (3) math marks in Elementary, Junior High, and High School.

The majority of the reported experiences in the open-ended questions did not relate to MA. In the Support category, reporting that someone did something to decrease math confidence was positively related to math anxiety, but this connection disappeared after controlling for general and test anxiety. Receiving help from parents, on the other hand, was related to lower MA above and beyond general and test anxiety. In regards to Instruction, MA was higher for individuals who experienced a teacher do something to increase their anxiety about math, and also when a teacher made them feel poorly about themselves, but these findings were no longer significant once general and test anxiety are considered. However, there was a significant decrease in MA when participants reported that their teachers provided plenty of examples and practice items, and this remained after controlling for general and test anxiety. For the Life Events questions, moving and changing schools during Junior High was related to higher MA, but this also became non-significant after controlling for general and text anxiety. Finally, doing poorly or below expectations in math were negatively related to MA even after considering general and test anxiety.

## Discussion

The purpose of the current study was to assess the potential relation between math anxiety (MA) and math experience. Specifically, the study was designed to investigate this relation with MA within four general themes: Support, Instructional Methods, Life Events, and Math Marks. The following section will review and expand on the results for each of these themes in turn.

### Lack of support

The first hypothesis focused on the relation between MA and perceived level of support. It was hypothesized that individuals who perceived having lower levels of support in Elementary, Junior High, and High School would have higher levels of MA than those who perceived higher levels of support. For the Likert-items, this hypothesis was only partially supported as the relation only existed during High School. This negative relation is consistent with research reporting higher MA (Baloglu and Koçak, 2006) and higher math test anxiety (Gierl and Bisanz, 1995) in older students compared to younger students. It could be that students receive adequate support in their younger schooling years and this support wanes as individuals advance in education. It may also reflect the fact that earlier level math is less demanding than High School math, and therefore the need for increased support is more critical in High School. It is also possible that support experiences from High School are fresher in the minds of participants, given that these experiences are more recent, and it is this increased memory accuracy that makes this relation stronger. There is no literature that supports these suppositions, so they would be worthwhile to examine in future research.

In the open-ended questions, the majority of participants (88%) reported that someone did something to increase their math confidence and 60% reported having someone do something to decrease their confidence in math. Nevertheless, MA was not related to reporting that someone increased your math confidence, even after accounting for general and test anxiety. Participants stated a number of teacher variables (e.g., teacher treated me poorly, teacher was critical) that they believed were associated with their levels of MA, however, no differences were found between these variables. Other studies suggest that having insensitive, hostile, and critical teachers (Jackson and Leffingwell, 1999), and teachers that use disrespect, humiliation, and fear-based instruction (Schmidt, 2005) is related to higher levels of MA, but we did not find evidence to support this claim. Our findings may diverge from this previous research because these researchers used sample known to be highly math-anxious participants, while we used participants with varying levels of MA.

There was one specific action that did have a positive impact on MA. Receiving help from a parent was related to lower MA. This finding is consistent with Schmidt's (2005) inverse finding that disbelieving or abusive parents were related to higher MA, although the presence of parental help is not the same thing as the absence of parental abuse. Focusing on our positive finding, it is possible that having parents willing to help may provide an environment conducive to reducing MA. It may also provide a model for how to approach math learning in a non-anxious way. On the other hand, it is possible that parents who are more willing and are able to help with math also happen to be better at math, and this difference in parental math ability would be related to the participant's ability. In any case, parental help in math is a factor that warrants further examination.

It is interesting to note that the High School Support scale was related to MA even though almost none of the specific examples mentioned in the open-ended questions exhibited the same relation. This is even more striking after observing that, given the scale was about different stages of schools, the items in the support scale are mostly about teacher support while none of the open-ended responses regarding teachers were related to MA. It is possible that support has a more subtle or cumulative influence on MA such that particular events may not stand out or come to mind when asked about them in an open-ended fashion. If, however, someone is prompted to respond to Likert-items that ask about particular support scenarios, then their response may reflect a more subtle and consistent environment that is actually more related to the development of MA. This is another question that requires further research.

### Instructional methods

Instructional methods have been the most researched in terms of its impact on an individual's level of MA (Harper and Daane, 1998; Jackson and Leffingwell, 1999; Brady and Bowd, 2005). It was hypothesized that individuals who reported more negative experiences with instructional methods at each school level would have higher levels of MA compared to those who reported fewer negative experiences. With the Likert-items, this hypothesis too was only partially supported; instructional methods in Elementary and High School were negatively related to MA. These results are mostly consistent with studies conducted in this area, as the most common finding in the literature on instruction suggests poor instructional methods are related to increased MA (Harper and Daane, 1998; Jackson and Leffingwell, 1999; Brady and Bowd, 2005), although there are no findings to suggest why this relation may not be as relevant for Junior High compared to Elementary and High School.

Results from the open-ended questions, however, provide little support for the hypothesis. There were no significant differences in MA between those who did and did not have a teacher increase or decrease their MA. Previously identified instructional methods related to increased MA include embarrassing students in front of their classmates, gender bias, having a negative attitude, responding angrily when asked for clarification, etc. (Jackson and Leffingwell, 1999; Brady and Bowd, 2005). Although our sample reported similar events (e.g., teacher was unapproachable, teacher was angry or frustrated, and the teacher spoke about how hard math was), we did not find a difference in MA between those who did and did not report these events. These results were somewhat surprising given the findings of the existing literature (Jackson and Leffingwell, 1999; Brady and Bowd, 2005; Bonnstetter, 2007), but this may again be due to having a wider range of math anxiety in our study.

On a positive note, those who reported having a teacher give plenty of examples had lower MA compared to those who did not. This is promising evidence from a treatment perspective, as one of the main components for the treatment of anxiety disorders is exposure (Craske and Barlow, 2008). Anxiety management techniques are currently used to reduce MA (Hembree, 1990) and math performance can be improved by controlling negative emotions and thoughts associated with math (Maloney and Beilock, 2012). Therefore, teachers who provide plenty of examples to their students are exposing them to more math, which may help them face their fears and habituate to the anxiety they experience with math.

Similar to the inconsistency found with the Support category, there was a largely present relation between Instructional Methods and MA on the Likert-items, but practically no relation with MA on the open-ended items. As with the Support scale, it is possible that the Likert-items are reflecting a more cumulative and overall effect of good instructional practice. Thus, it may be generally good instructional practice, and, with the notable exception of providing many examples and practice tests, not specific instructional practices, that is related to lower MA. Alternatively, it may be that the specific practice of providing many examples is what is driving the relation with MA, and the instructional method subscale is indirectly reflecting experience with teachers who are more likely to do this.

### Life events

The relation between life events and MA was the only theme assessed primarily by open-ended questions. It was hypothesized that individuals who reported negative life events during their school years would have higher levels of MA compared to those who did not experience these events. This hypothesis was not supported as we found virtually no differences in MA for those who experienced negative life events and those who did not. The one exception to this was higher MA for those who reported doing poorly or below expectations in school, which is not surprising.

Initially, it appeared that MA was related to moving homes or moving schools during Junior High; however, once general and test anxiety were controlled for, this relation disappeared. Previous work suggests that MA is related to moving schools (Zopp, 1999). However, Zopp's participants were adult learners with high MA, and the role of general and test anxiety was not considered. Moreover, she did not assess moves during different educational periods. We did not find moving schools or homes to be predictive of the level of MA experienced by students in Junior High, but it may be that moves at this time can heighten general anxiety. Junior High coincides with the transition into adolescence, with its accompanying changes in physical appearance and emotional well-being. It is possible that these changes could result in self-doubt that transfers into academics. For this reason, Junior High could be a more vulnerable time than Elementary and High School, where moving schools may bring about difficulties that could lead to increased general anxiety as well as MA.

It is also possible that some of the other life events that had trending differences in MA (e.g., having a family member experience mental or physical problems) may also relate to heightened general anxiety, but not MA specifically. However, most of the life events reported did not demonstrate a trend toward differences in MA, contrary to the findings of previous research (i.e., Zopp, 1999; Schmidt, 2005). It is possible that participants' retrospective responses were unreliable, or due to social desirability. Perhaps providing information about abusive parents, turbulent home life strife with substance abuse, and mental health problems were not events participants were willing to discuss, even in an anonymous questionnaire. Having said that, many participants did report mental health issues and substance abuse issues (no one reported abusive parents), but it is possible that the people who would have been most affected by it (which may have influenced their MA) did not report these problems. Or, it just could be that these kinds of life experiences are not related to MA.

### Math marks

It was hypothesized that individuals who had lower math marks would have higher levels of MA compared to those with higher math marks. This hypothesis was fully supported, as our results indicate that math marks were negatively associated with MA during all three time periods.

Not only are the results consistent with previous research (Hunsley and Flessati, 1988; Schmidt, 2005), the relation between MA and math marks was the strongest of four themes examined. To be more precise, our findings are strictly that MA is negatively related to retrospectively recalled, self-reported, and unverified math marks. Caution needs to be drawn when interpreting these results: Is anxiety causing poor math marks, or are poor math marks causing anxiety? This question would be difficult to examine, as it is possible that MA is interfering with concentration and memory when learning math, but it is also possible that poor marks could decrease an individual's confidence in math, in turn creating MA. This question cannot be answered by the current study, but would be a worthwhile topic for future research.

### Limitations and future directions

Even though the results of this study are suggestive, there are limitations that should be kept in mind when interpreting these results. First, this study is correlational, so we cannot say if the math experiences reported by participants caused their MA, or if their MA is affecting the way they recall and interpret experiences. Future research will have to examine if changes in the factors identified above (e.g., if teachers introduce more examples or practice items) will lead to changes in math anxiety.

Second, this study used a retrospective self-report measure to assess math experiences, so inaccurately reported experiences are possible. Anxious individuals have a tendency to recall and perceive information in a negative manner because of their tendency to attend more to negative events regardless if positive things occurred, and are prone to perceive situations which are unclear in a negative threatening manner (Aikins and Craske, 2001). Therefore, information recalled by anxious individuals may be unknowingly reported in a distorted manner. Self-report data is also susceptible to social desirability, meaning that individuals may have responded to the questions in the manner in which they thought was desired by the researcher. Furthermore, students who are high in Math Anxiety may be more likely to recall negative experiences in math that other non-math anxious students also experienced but did not recall. Although these are all valid concerns, it is worthwhile to note that the pattern of results reported above does not reflect what these retrospective biases might predict. If highly math anxious people were more likely to report comparatively more negative experiences because of their anxiety shaping their memories rather than because of their actual experiences, then it seems reasonable that they would be just as likely to report more negative events for the open-ended questions as they would be to bias their Likert responses. Instead, almost none of the responses to the open-ended questions ended up being related to math anxiety, while the Likert scales did show relatively stronger results. It is possible that, in general, it was more difficult for participants to talk about their negative experiences in the open-ended questions, but it not clear how biases from retrospective reporting could lead to this result. Furthermore, the few significant results from the open-ended questions do not lend themselves to a retrospective explanation. It is not clear why somebody who is more math anxious would erroneously remember less parental help but not less parental praise or more parental criticism. Likewise, it not clear why less math-anxious individuals would be more biased to recall being given more examples or practice items by their teachers, but not more often recall their teachers offering praise, encouragement, or being available to help. Biases from the retrospective reporting of these results may still be affecting the results, but the data does not match the expected pattern if they were only due to these biases.

This study also developed a new measure of math experiences, and although we examined internal reliability of inter-rater reliability, this measure can be improved through further psychometric testing. Furthermore, by being thorough and surveying a wide range of math experiences, while also have stringent Type I error control, we may have also increased the probability of Type II error, especially when examining the open-ended questions. Some of these comparisons may not have had sufficient power, especially when one of the groups in the comparison was very small. This means that some effects, especially smaller effects, may have been missed.

Finally, the sample in this study was limited to university students, a large number of whom were Psychology majors. Many of these students may have recently experienced math anxiety by taking stats courses, and that might affect how they recall their math experiences. Furthermore, adults who do not go on to university might have different kinds of experiences that relate to math anxiety that would not be captured in these data. Nevertheless, our sample also consisted of a fair proportion of Science students, and overall we had a good range of math anxiety, which gives us some confidence in our ability to generalize, at least to other university students.

Many of these limitations, however, provide opportunities for future research. Potential problems with retrospective biases could be best resolved by conducting a similar study developmentally. By asking about (or recording) experiences as they happen, one could test whether we would find the same pattern of results. It would be quite onerous to ask about all possible events while they happen, and to be able to follow children long enough in time to see the effects of those events, but specific results of this study could be targeted for testing. For example, a future study could rate classroom teaching practices during the year and see if they relate to MA changes over that same year. Future research in this field should also include a measure of social desirability to assess its impact on the obtained results. Refining the measure of math experiences presented in this study and subjecting it to further psychometric testing would be beneficial to this research area. More focused research on specific themes could also validate, replicate, and test to see if any other math experiences are related to math anxiety.

## Conclusions

The goal of this study was to address a gap in the literature regarding the relationship between math experiences and math anxiety (MA), specifically those of support, instructional methods, life events, and math marks. This is the first study to systematically assess these four variables in relation to MA. It demonstrated the importance of support from teachers and parents where math is concerned, as well as the importance of support in High School. This is helpful as it may serve as a manner of prevention or reduction of MA. Moreover, the importance of “good” instructional methods was shown. Specifically, participants who said they had a teacher who provided plenty of examples had less MA. More work is needed to further assess what variables are considered to be “good” instructional methods that alleviate MA. This information is invaluable and could lead to the implementation of improved instructional methods into the school system, perhaps reducing MA in the process. Overall, the results of this study can be considered the first step in addressing a large gap within the literature of MA.

This study may also provide useful information for individuals working in both the educational and potentially the mental health systems. With regards to the educational system, it is important for teachers to be aware of factors that contribute to MA in an effort to help with its reduction and prevention. Moreover, it is important for teachers to be able to recognize when or if their students are experiencing MA so that the proper interventions can be put in place (i.e., strategies for anxiety management). Individuals working in mental health may also benefit from further information with regards to MA to effectively create treatment programs for those who are affected by this specific anxiety. At the same time, this study demonstrated that many different kinds of experiences, thought to be related to MA, may not be related after all. This is also an important finding, as it may prevent resources from being directed on improving experiences that are not actually related to MA.

## Ethics statement

This research was approved by the Interdisciplinary Committee on Ethics in Human Research (ICEHR) committee at the host university. The primary researcher of the current study sent an email to several of the faculty members within the psychology department at the host university. This email provided detailed information on the current study and its purpose, along with a request for professors' permission to allow the researcher to enter into their undergraduate class to explain the study to the students in an attempt to recruit participants. Once permission was granted the researcher scheduled a date with the professor to come and explain the study to the students. During the classroom visit the researcher or her assistant read aloud a standardized script to the students describing the study and its purpose and answered any questions that arose. At the end of the explanation the researcher or the researcher's assistant informed all students that participation in this study was voluntary and that if they agreed to participate they had the freedom to withdraw at any point in time. Additionally, an email outlining the study was sent to each student in the class along with possible times that they could participate. During the data collection session the researcher provided the students with an information letter explaining the study and its purpose. The participants were informed that their participation was voluntary and that they could withdraw from the study at any point without fear of being penalized. After going through the information and agreeing to complete the study, the students were asked to sign the consent form if they wanted to participate in the study. Following the completion of the consent form, the participants were asked to complete the questionnaire packet containing the Math Anxiety Rating Scale-Short Version (MARS-S), the Math Experience Questionnaire, the 50-item International Personality Item Pool (IPIP) representation of Costa and McCrae's (1992) five NEO domains (Goldberg, 1999), the Test Anxiety Inventory (TAI), the Penn State Worry Questionnaire (PSWQ), the Beck Anxiety Inventory (BAI), and a demographics questionnaire. Upon completion of the questionnaire packet, participants were given a debriefing form, were asked if they had any questions, and were thanked for their participation. Participants were told that if they wished to see the results of the overall study, they could contact the researcher.

## Author contributions

This manuscript represents the thesis work of KO. KO primarily designed the study, collected the data, conducted the data analyses, and wrote the primary manuscript. CF assisted in data collection and helped in editing and data analysis. DH was the supervisor on the project and contributed to study design as well as editing the manuscript.

### Conflict of interest statement

The authors declare that the research was conducted in the absence of any commercial or financial relationships that could be construed as a potential conflict of interest.
