# Supplementary material for: Math Anxiety Is Related to Some, but Not All, Experiences with Math
Source: Front Psychol. 2017 Dec 1;8:2067. doi: 10.3389/fpsyg.2017.02067 (PMC5770635; doi:10.3389/fpsyg.2017.02067)
Supplement: Supplementary file 1 [file DataSheet1.docx]

# Appendix A

# Likert-items on Math Experience Questionnaire

This questionnaire was given three times, one for each level of schooling (e.g., Elementary, Junior High, and High School). Each item was ranked on a 5-point scale from Strongly Disagree to Strongly Agree.

| Support Items | |
| --- | --- |
| 1. | My math teachers encouraged me to do well in math |
| 2. | I could turn to my teachers when I needed help understanding math concepts |
| 16. | My math teachers were understanding when I asked for help with math |
| 18. | I was frequently left alone to work on math problems* |
| Instructional Methods Items | |
| 4. | My math teachers acted as though what they were teaching was easy to learn* |
| 5. | My math teachers were kind |
| 6. | My teachers taught math too quickly for me to grasp the important concepts |
| 7. | My teachers tried to make math fun |
| 9. | My teachers encouraged me to try new methods to solve math problems |
| 10. | My math teachers frequently had us work through math problems in groups |
| 11. | My teachers taught math rules without teaching the theory behind them |
| 12. | My math teachers were patient |
| 13. | My math teachers ensured that they explained the terminology they were using |
| 14. | My teachers tried to connect math to everyday life |
| 17. | My teachers frequently asked me to answer questions in front of the class (i.e. on the black board, out loud)* |
| 19. | My math teachers were critical* |
| 21. | My math teacher frequently employed math competitions or games to help with teaching math concepts* |
| Math Marks Items | |
| 8. | My math marks were lower compared to other subjects |
| 22. | My math marks were higher compared to other subjects |
| 24. | My math marks were not up to my expectations |
| Miscellaneous | |
| 3. | My math teachers seemed to favor boys over girls |
| 15. | My math teachers had higher expectations for boys than girls |
| 20. | My math teachers frequently allowed us to use a calculator when working on math problems |
| 23. | It was obvious that my teachers did not like math |

Note: Items with an asterisks were eliminated from the final scale.

Appendix B

# Open-ended Items and Codes for Math Experience Questionnaire

**1. In your past did anyone (i.e. a teacher, parent, or peer) do something to increase your confidence in your math ability? Please explain.**

*Codes:* Extra help from teachers; Extra help from parents; Extra help from tutors; Encouragement, Support, or Praise from teachers; Encouragement, Support, or Praise from parents; Encouragement, Support, or Praise from tutors; Teachers personality (Kind, Caring, Understanding, Want students to do well); Being involved or being told you should be involved in an advanced or enrichment program; Being asked to be involved in Math competitions or leagues; Being asked to tutor others; Other

*Interrater Percentage Occurrence Agreement*: 66.7%

**2. In your past did anyone (i.e. a teacher, parent, or peer) do something to decrease your confidence in your math ability? Please explain.**

*Codes:* Lack of encouragement, praise, support and help; Math teacher was unapproachable; Math teacher made me feel poorly about myself (i.e. Made me feel stupid, were belittling and/or laughed at me); Math teacher was not concerned for how well people did; Thinking negatively about one’s own abilities; Peers made fun of those that did poorly; Peers made fun of me because I did well; Parents would become frustrated and angry when teaching me math; Teachers would become frustrated and angry when teaching me math; My teacher hated math; My friends did better than me; There was negative feedback on tests when I got it back; I had high expectations for myself; Others had high expectations for me; Other

*Interrater Percentage Occurrence Agreement*: 91.2%

**3. Did your teacher do anything to increase your anxiety about math? Please explain**

*Codes:* Teacher was unapproachable; Teacher was angry or frustrated; Teacher spoke about how difficult math was; Emphasis placed on test and/or doing well on them; Teachers moved through material too fast; Testing on material that was not covered in class; My teacher had high expectations; Math teacher made me feel poorly about myself (i.e. Made me feel stupid, were belittling and/or laughed at me); Other

*Interrater Percentage Occurrence Agreement*: 64.9%

**4. Did your teacher do anything to decrease your anxiety about math? Please explain.**

*Codes:* Teacher explained and/or answered question until they were understood; Encouragement, Praise, Support; Weekly math quiz’s on material learnt; Teacher was patient and/or Understanding; Teacher made math fun and/or simple; I did well in math; Teacher was available for extra help (i.e. they were helpful, gave tutorials); Gave lots of examples and/or practice tests; Other

*Interrater Percentage Occurrence Agreement*: 79.2%

**5. How many times did your family move homes when you were a child? During what grades did these moves occur (Elementary School, Junior High School or High School)?**

*Codes:* Elementary School; Junior High School; High School; Moved however, it was prior to elementary school; Moved however, no grade was indicated

*Interrater Percentage Occurrence Agreement*: 100%

**6. How many times did you have to change schools other than the transitions made from elementary to junior high school and junior high to high school? During which grades were these moves made?**

*Codes:* Elementary School; Junior High School; High School; Moved however, it was prior to elementary school; Moved however, no grade was indicated

*Interrater Percentage Occurrence Agreement*: 100%

**7. Have you ever had to move to a new school within a school year? If so, in what grades?**

*Codes:* No; Kindergarten; One; Two; Three; Four; Five; Six; Seven; Eight; Nine; Ten; Eleven; Twelve

*Interrater Percentage Occurrence Agreement*: 83.3%

**8. Did moving schools affect your academic performance in math? Please explain.**

*Codes:* No; Yes, my marks improved; Yes, my marks got worse; Yes, it was hard to adjust to new surroundings, teachers, peers and curriculum; Yes, things got worse; Yes, other

*Interrater Percentage Occurrence Agreement* 78.0%

**9. What events at home stand out for you in shaping your feelings about math? Please explain.**

*Codes:* Parents help; Parents encouragement and praise; Sibling Rivalry; Parents stressing that math was important and to do well; Parents stressing that it was important to do well overall; Routine of practicing math; Helping siblings with math; Parents made me feel bad and/or punished me for low marks in math; Having parents or family members that were good at math; Other

*Interrater Percentage Occurrence Agreement*: 76.3%

**10. What events in school stand out for you in shaping your feelings about math? Please explain.**

*Codes:* Good math teachers (i.e. teachers that are encouraging, supportive and helpful); Bad math teachers; The manner in which you were treated depending on what stream you were doing (advanced, academic or basic); Being asked to take part in competitions and/or clubs; Doing well and/or being confidant in my math abilities; Getting awards or being nominated for awards in math (i.e. winning math competitions); Being in advanced math; Doing poorly or lower then my expectations in math; Other

*Interrater Percentage Occurrence Agreement*: 74.2%

**11. Did you have any positive or negative experiences in math-related to your gender? Please explain.**

*Codes:* Positive; Negative

*Interrater Percentage Occurrence Agreement*: 92.3%

**12. During your time at school did you or a family member experience any major physical or mental health problems?**

*Codes:* No; I experienced Mental health problems; I experienced physical health problems; I experienced both; I experienced neither; My family experienced Mental health problems; My family experienced physical health problems; My family experienced both; My family experienced neither; Other (i.e. Deaths); Yes, however, no specifics given

*Interrater Percentage Occurrence Agreement*: 88.6%

**13. During your time at school did you or a family member experience substance abuse problems?**

*Codes:* No; Yes I experienced substance abuse problems; No I did not experience substance abuse problems; Yes my family experience substance abuse problems; No my family did not experience substance abuse problems; Yes, however, no specifics given

*Interrater Percentage Occurrence Agreement*: 100%

**14. Did you experience anything in your personal life that you believe affected your academic ability? Please explain.**

*Codes:* Substance abuse; Mental health problems (personal or family member); Interpersonal Problems; Physical health problems (personal or family member); Transitions from high school to university; Being made fun of my peers; Parents separation or divorce; Death of family members; Lack of self confidence; Too many extra circular activities; Other

*Interrater Percentage Occurrence Agreement*: 100%
